# Predictors of Wound Complications following Radiation and Surgical Resection of Soft Tissue Sarcomas

**DOI:** 10.1155/2017/5465130

**Published:** 2017-06-15

**Authors:** Drake G. LeBrun, David M. Guttmann, Jacob E. Shabason, William P. Levin, Stephen J. Kovach, Kristy L. Weber

**Affiliations:** ^1^Perelman School of Medicine, University of Pennsylvania, Jordan Medical Education Center, 3400 Civic Center Blvd., Philadelphia, PA 19104, USA; ^2^Department of Radiation Oncology, Perelman School of Medicine, University of Pennsylvania, 3400 Civic Center Boulevard, TRC 2 West, Philadelphia, PA 19104, USA; ^3^Department of Plastic Surgery, Perelman School of Medicine, University of Pennsylvania, 3400 Civic Center Boulevard, 7th Floor South Pavilion, Philadelphia, PA 19104, USA; ^4^Department of Orthopaedic Surgery, Perelman School of Medicine, University of Pennsylvania, 3400 Civic Center Boulevard, 10-179 South Pavilion, Philadelphia, PA 19104, USA

## Abstract

Wound complications represent a major source of morbidity in patients undergoing radiation therapy (RT) and surgical resection of soft tissue sarcomas (STS). We investigated whether factors related to RT, surgery, patient comorbidities, and tumor histopathology predict the development of wound complications. An observational study of patients who underwent STS resection and RT was performed. The primary outcome was the occurrence of any wound complication up to four months postoperatively. Significant predictors of wound complications were identified using multivariable logistic regression. Sixty-five patients representing 67 cases of STS were identified. Median age was 59 years (range 22–90) and 34 (52%) patients were female. The rates of major wound complications and any wound complications were 21% and 33%, respectively. After adjusting for radiation timing, diabetes (OR 9.6; 95% CI 1.4–64.8; *P* = 0.02), grade ≥2 radiation dermatitis (OR 4.8; 95% CI 1.2–19.2; *P* = 0.03), and the use of 3D conformal RT (OR 4.6; 95% CI 1.1–20.0; *P* = 0.04) were associated with an increased risk of any wound complication on multivariable analysis. These data suggest that radiation dermatitis and radiation modality are predictors of wound complications in patients with STS.

## 1. Introduction

The current standard of care for local control of disease in patients with soft tissue sarcoma (STS) of the extremities and trunk where an adequate margin cannot be obtained with resection alone is surgical resection combined with radiation therapy (RT). The use of RT is also preferred in patients with deep, high-grade, large tumors regardless of the ability to achieve adequate margins. However, multiple studies have demonstrated overall wound complication rates ranging from 22 to 35% when radiation was given in combination with surgery [1–4], with even higher rates seen when individual limbs were assessed. In fact, a large randomized control trial found a wound complication rate of 45% in patients with STS of the thigh [4].

Wound complications are treatable with good long-term functional status, yet they can be sources of substantial morbidity in patients with STS. Major wound complications encompass a broad array of wounds including those that require reoperation, an invasive procedure without general or regional anesthesia, readmission for wound management, or prolonged deep packing or dressing changes [4]. In contrast, minor wound complications—those that necessitate clinical follow-up but do not fit within the scope of major wound complications—are also clinically important though not as frequently reported in the literature. To reduce the risk of wound complications, it has been standard practice at sarcoma centers to perform soft tissue reconstruction with the use of rotational or free flaps to bring viable muscle or fasciocutaneous tissues to the defect left after tumor resection. Despite these efforts, wound complications remain a significant problem in this population.

Although RT is known to increase the risk of wound complications, there is limited available data on radiation-related factors other than radiation timing that may be predictive of wound complications in the setting of RT and surgical resection of STS. In addition, further work is needed to refine our understanding of baseline demographic factors that may predispose patients to wound complications. We therefore sought to identify predictors of wound complications in this population with a specific focus on radiation-related parameters that have not been extensively studied in the literature. We hypothesized that factors associated with radiation technique would be correlated with the development of wound complications after adjusting for established clinical risk factors.

## 2. Materials and Methods

### 2.1. Patient Population

After obtaining approval from our Institutional Review Board, we identified all patients who had primary resection or reresection of biopsy-proven STS at our institution between August 2013 and November 2016. Patients were excluded from analysis if they underwent resection for regionally recurrent disease, were less than 18 years old, did not undergo RT, or had retroperitoneal tumors. All surgical resections were performed at one institution. Use of muscle, fasciocutaneous, and/or skin grafts was anticipated preoperatively with surgical planning between an orthopaedic oncologist and a plastic surgeon. Indications included a substantial skin or soft tissue defect that could not be closed primarily or a need for vascularized tissue over exposed bone, joint, or neurovascular structures. Radiation was delivered at our institution or at outside institutions. For the six patients who underwent RT at outside institutions, radiation reports were obtained from the patient's primary radiation oncologist. These reports included all relevant radiation parameters including modality and the presence of acute skin toxicities. If any parameters were missing or unclear, these were confirmed via contact with the patient's radiation oncologist. Generally, patients with intermediate- or high-grade tumors or tumors larger than 5 cm underwent RT. All patients are followed routinely in the clinic and there was no loss to follow-up over the study period.

### 2.2. Data Collection

Patient, tumor, and treatment-related data were collected by retrospective review of the medical record. Patient data included patient age, sex, obesity (defined as BMI ≥ 30), diabetes status, and smoking history. Diabetes status included both Type I and Type II diabetes mellitus.

Tumor data included resected specimen size, tumor size, tumor depth relative to the fascia, and tumor location. Resected specimen size and tumor size were defined by the maximal cross-sectional diameters of the total resected specimen and tumor, respectively, as indicated in the pathology report. Tumor depth was defined as either deep or superficial relative to the fascia. Tumors that were both deep and superficial to the fascia with subcutaneous extension were considered superficial for the purposes of data analysis.

Treatment-related data included history of chemotherapy, type of wound closure, radiation timing (pre- versus postoperative), radiation modality, grade of radiation dermatitis, use of radiation boost, use of radiation bolus, planning target volume (PTV), and cumulative radiation dose. Radiation dermatitis was a dichotomous variable (grade <2 versus grade ≥2) determined by the radiation oncologist's summary report and based on the 2010 National Cancer Institute Common Terminology Criteria for Adverse Events [5]. Radiation boost is defined as the escalation of the total radiation dose prescription to a subvolume of the initial treatment region believed to be at higher risk for local recurrence. Radiation bolus is the use of tissue equivalent material placed over the treatment region to increase dose deposition in more superficial structures such as skin. PTV is the total volume of tissue targeted to receive the prescription dose of radiation.

### 2.3. Outcomes

Major and minor wound complications were assessed up to four months postoperatively. Major wound complications were defined according to the National Cancer Institute of Canada trial as a reoperation for wound repair, an invasive procedure for wound management without regional or general anesthesia, hospital readmission for nonoperative wound management, or prolonged deep packing or dressing changes for 120 days or longer [4]. Minor wound complications were defined as any other surgical wounds that did not meet criteria for a major wound. These included wounds requiring in-office debridement or oral antibiotics. A prespecified composite outcome “any wound” was defined as the development of either a major or minor wound complication. The time interval between surgery and first identification of a wound complication was also recorded.

### 2.4. Statistical Analysis

Descriptive statistics were used to summarize demographic, histopathologic, and treatment characteristics of the study population. Fisher's exact test and the Wilcoxon rank-sum test were used to determine whether categorical and continuous characteristics were associated with the development of wound complications, respectively. Due to the limited number of events in this dataset, a parsimonious model was desired to avoid model overfitting. A backward selection strategy was used with a threshold to enter *P* < 0.2 to screen weak prognostic covariates from the multivariable model. Variables were retained in the multivariable model if Wald tests yielded a *P* value of <0.05. Generalized estimating equations (GEE) were used to adjust for correlated exposures and outcomes among individuals with multiple observations in the sample [6]. Collinearity was assessed by calculating variance inflation factors and the final model was evaluated for two-way interactions. Model goodness-of-fit was assessed via the Hosmer-Lemeshow test. Discrimination—the ability of our model to assign higher probabilities of wound complications to those with true wound complications compared to those without—was assessed via the *C*-statistic, also known as the area under the receiver operating curve [7]. Fewer than 5% of observations were missing for any variable included in the regression analysis. For all analyses, two-sided tests were used with a level of significance of *α* ≤ 0.05. All analyses were performed using STATA 14.0 (StataCorp, College Station, TX, USA).

## 3. Results

Patient and tumor characteristics are summarized in Table 1. A total of 65 patients met inclusion criteria for the study. Two patients had separate resections and radiation treatments in the setting of metastatic disease and were each considered as two distinct cases for the purposes of this study for a total of 67 cases. The median age of patients was 59 years (range 24–90 years) and 34 (52%) of patients were female.

Treatment characteristics are summarized in Table 2. Among preoperative RT patients, resection occurred a median of 28 days (range 18–49 days) after completion of RT. Surgery for patients who underwent reresection occurred between 25 and 162 days after their primary resection at an outside hospital. All patients underwent limb-salvage resection, and all patients were alive at the 120-day cutoff to assess wound complications. With a median postoperative follow-up of 20.7 months, 60 patients (92%) were alive at the time of the study analysis.

A summary of wound types is shown in Table 3. Among all cases, 14 (21%) major wound complications were noted within four months of surgery. An additional 8 (12%) minor wound complications were noted; thus, a total of 22 cases (33%) experienced* any* wound complication. Wounds were first documented a median of 30 days postoperatively (range 11 to 100 days).

On univariate analysis, only diabetes was associated with the development of a major wound complication (*P* = 0.05). Diabetes (*P* = 0.01), skin graft and/or vascularized flap reconstruction (*P* = 0.01), grade ≥2 radiation dermatitis (*P* = 0.02), and 3D conformal RT relative to intensity-modulated radiation therapy (IMRT) or proton therapy (*P* = 0.008) were associated with an increased risk of any wound complication.

Among patients undergoing preoperative RT, 7 of 13 (54%) with grade >2 radiation dermatitis developed any wound complications compared to 11 of 43 (26%) without high-grade radiation dermatitis. Although there was evidence of an association among this subgroup, it did not meet statistical significance (OR 3.39; 95% CI 0.94–12.30; *P* = 0.06). Among patients undergoing postoperative RT, 3 of 5 (60%) with grade >2 radiation dermatitis developed any wound complications compared to 1 of 4 (25%) with grade <2 radiation dermatitis (OR 4.50; 95% CI 0.25–80.57; *P* = 0.31).

No patients who underwent proton therapy (*n* = 10) developed wound complications. When proton therapy was excluded, the use of 3D conformal RT was still associated with more wound complications relative to IMRT (*P* = 0.04). Tumor depth did not significantly affect any wound development, with any wounds seen in 25% of those with deep tumors and 45% of those with superficial tumors (*P* = 0.09).

In the multivariable model, diabetes remained associated with an increased risk of major wounds (OR 5.10; 95% CI 1.07–24.29; *P* = 0.04). For any wounds (Table 4), diabetes (OR 9.58; 95% CI 1.42–64.83; *P* = 0.02) and grade ≥2 radiation dermatitis (OR 4.82; 95% CI 1.20–19.21; *P* = 0.03) remained associated with an increased risk of wound complications. The use of 3D conformal RT was also found to be significantly associated with an increased risk of wound complications relative to IMRT or proton radiation (OR 4.55; 95% CI 1.09–20.0; *P* = 0.04). Radiation timing was included in the multivariable logistic regression model to account for known differential risk associated with pre- versus postoperative RT; however, it was not associated with wound complications in either model. The *C*-statistic was 0.80 suggesting good ability of the model to discriminate between individuals who did and did not experience any wound complication. The Hosmer-Lemeshow test demonstrated adequate goodness-of-fit. No collinearity was detected and no significant interactions were identified.

## 4. Discussion

In this single-center observational study of 67 cases of STS of the extremity and trunk that underwent surgical resection and RT, we found that 3D conformal RT was associated with a greater risk of wound complications relative to more conformal modalities such as IMRT or proton therapy. Moreover, we found that grade ≥2 radiation dermatitis was associated with an increased risk of wound complications, a finding that has not been identified in other studies.

Radiation therapy is an integral component of limb-sparing treatment for most patients with STS and allows marked improvement in local control compared to surgical resection alone [8]. However, the addition of RT is not without risks. Wound complications are a source of considerable morbidity for these patients. Although much of the existing literature has focused on major wound complications, we sought to expand this definition given the occurrence of other wound complications that are clinically important but not accounted for in previous studies [4]. In doing so, we sought to more precisely establish the risk factors associated with the development of major wound complications and any wound complications, with an expanded focus on radiation parameters and outcomes.

To our knowledge, this exploratory study is the second to show that the use of 3D conformal RT was associated with a greater risk of wound complications relative to more conformal modalities such as IMRT or proton therapy. Our findings are consistent with a recent study by Saeed et al. [9] that demonstrated a decreased risk of postoperative wound complications (OR 0.4, *P* = 0.02) among patients undergoing IMRT compared to patients who underwent 3D conformal RT for STS. Otherwise, a 2013 study showed that the rate of wound complications using IMRT was lower than that of the landmark NCI study that used 3D conformal radiation but failed to meet statistical significance [10]. A recent prospective phase II trial assessed the use of image-guided RT with reduced volumes, with all patients undergoing preoperative RT and 75% receiving IMRT [11]. This study demonstrated a similar acute wound complication rate (36.6%) to the National Cancer Institute of Canada trial with a lower rate of late toxicities. Importantly, these studies had larger sample sizes and were better powered than the current study [10, 11]. Results of the prospective phase II trial Preoperative Radiotherapy for Sarcomas of the Extremities with Intensity-Modulation, Image-Guidance, and Small Safety-Margins (PREMISS) are awaited to clarify whether IMRT can reduce wound complications in this population [12].

There is a pressing need for additional studies to corroborate the recent findings by Saeed and colleagues [9]. Our study addresses that need by providing further evidence to support the association between radiation modality and wound complications. Compared to 3D conformal RT, IMRT and proton radiation permit more conformal targeting of complicated volumes, sparing normal tissue from excess dose and decreasing associated toxicity [13]. 3D conformal radiation techniques may deliver a higher dose to a larger volume of the skin and subcutaneous tissues, placing the patient at higher risk for wound breakdown. This may be the basis for the observed association between 3D conformal radiation and wound complications. In contrast, IMRT limits the high dose radiation volume more tightly to the tumor by using multiple beam angles of varying fluency to deliver dose to the target. Proton therapy even better spares normal tissue due to the physical properties of protons that cause them to deposit all their energy in the target tissue and completely spare dose distally. This is distinct from traditional photon radiation in which there is always “exit” dose along the beam path. In this study, the small size precluded a reliable statistical comparison between IMRT versus proton therapy or proton therapy versus 3D conformal RT. Although our study was not designed to assess outcomes associated with proton therapy alone, no patients who underwent proton therapy developed wound complications, highlighting an area for further inquiry. From a practical perspective, it is not infrequent that insurance companies deny the use of IMRT or proton therapy for treatment of patients with soft tissue sarcomas, citing no evidence to justify its increased cost. The data in this manuscript may be helpful in obtaining authorization for these advanced treatments, as the risk of wound complications is reduced.

This study is also the first to identify grade ≥2 radiation dermatitis as an independent predictor of wound complications in the setting of STS. It is unclear if there is a direct causal relationship between radiation dermatitis and surgical wound complications or if the development of radiation dermatitis is a surrogate marker for a patient who is biologically more susceptible to radiation injury. Radiation incites an active inflammatory response in irradiated tissue, which is associated with poor wound healing. The observed association between brisk radiation skin reaction and wound complications in this cohort likely results from a larger biological effect of the radiation in the tissues of those patients who experienced worse dermatitis based on both technical (e.g., choice of radiation modality) and patient factors (e.g., diabetes). It is also possible that the soft tissues in these patients were predisposed to complications or poor cutaneous healing due to unstudied patient factors such as inadequate nutrition.

Our results confirm the importance of diabetes in the development of wound complications, a finding that has been demonstrated in other studies assessing wound complication risk in patients undergoing resection and RT for STS [14–16]. When treating patients with STS and comorbid diabetes, clinicians should consider strategies to lower the risk of wound complications such as communicating with the patient's primary care physician at the time of diagnosis to optimize diabetes control and monitoring fasting glucose levels over the course of preoperative RT. Although we did not routinely check hemoglobin A1c preoperatively, this is another strategy to stratify patients by their degree of chronic hyperglycemia.

There are important limitations to this study. The study is retrospective and therefore subject to selection bias. Although we attempted to control for any measured confounding with multivariable analysis, unmeasured factors not accounted for in the data set may bias the estimates of wound complication risk. For example, we do not know why certain patients received IMRT or proton therapy and others did not. In addition, the study is underpowered to detect all relevant risk factors for wound complications in multimodality therapy for STS. This may explain why some previously identified risk factors did not meet statistical significance in our study, including smoking history, obesity, specimen size, and preoperative radiation [4, 14–17]. Other known risk factors such as vascular involvement of the tumor and tumor volume were not assessed in our study [18, 19]. Baldini and colleagues found that tumor depth, defined as tumor distance less than 3 mm from the skin surface, was associated with wound complications after radiation [15]. Our study defined tumor depth relative to the fascia, considering deep tumors with superficial extension as superficial given the need to manage these superficial tumors to remove the subcutaneous margin, and found no association between tumor depth and wound complications. When these tumors were considered deep, the results of the analysis did not significantly change.

## 5. Conclusions

In this study, the use of IMRT or proton therapy was associated with a decreased risk of wound complications relative to the use of 3D conformal RT after adjusting for radiation timing. These data suggest that utilizing IMRT or proton therapy in place of 3D conformal RT could reduce the incidence of wound complications in patients with STS. Moreover, this study suggests that grade ≥2 radiation dermatitis is associated with an increased risk of wounds, highlighting a previously undescribed predictor of postoperative wound complications. Additional studies are needed to corroborate and elucidate these findings.

## Figures and Tables

**Table 1:** Patient and tumor characteristics.

Characteristic	Major wound complication			Any wound complication		
	Yes	No	*P*	Yes	No	*P*
*Sex*			0.95			0.55
Female	7 (50%)	27 (51%)		10 (45%)	24 (53%)	
Male	7 (50%)	26 (49%)		12 (55%)	21 (47%)	
*Age*	62.2 (19.5)	57.0 (16.3)	**0.19**	62.8 (17.7)	55.7 (16.3)	**0.08**
*Obese (BMI ≥ 30)*			0.75			**0.08**
Yes	5 (36%)	16 (30%)		10 (45%)	11 (24%)	
No	9 (64%)	37 (70%)		12 (55%)	34 (76%)	
*Diabetes mellitus*			**0.05**			**0.01**
Yes	4 (29%)	4 (8%)		6 (27%)	2 (4%)	
No	10 (71%)	49 (92%)		16 (73%)	43 (96%)	
*Tobacco use*			0.69			0.65
Current or past smoker	5 (36%)	22 (42%)		14 (64%)	26 (58%)	
Never smoker	9 (64%)	31 (58%)		8 (36%)	19 (42%)	
*Tumor location*			1.0			0.24
Upper extremity	2 (14%)	8 (15%)		5 (23%)	5 (11%)	
Lower extremity	12 (86%)	42 (79%)		17 (77%)	37 (82%)	
Trunk	0 (0%)	3 (6%)		0 (0%)	3 (7%)	
*Tumor location in extremity*			0.74			0.77
Proximal	11 (79%)	36 (68%)		17 (77%)	30 (67%)	
Distal	3 (21%)	14 (26%)		5 (23%)	12 (27%)	
Trunk	0 (0%)	3 (6%)		0 (0%)	3 (6%)	
*Tumor depth relative to fascia*			0.83			**0.09**
Deep	8 (57%)	32 (60%)		10 (45%)	30 (67%)	
Superficial	6 (43%)	21 (40%)		12 (55%)	15 (33%)	
*Resected specimen size (cm*)^*∗*^	13.0 (13.7)	11.9 (13.1)	0.65	12.9 (8.7)	13.3 (6.4)	0.31
*Tumor size (cm)* ^*∗*^	11.1 (9.6)	7.9 (4.7)	0.60	10.1 (7.6)	7.8 (5.0)	0.49

^*∗*^Continuous variables are reported as mean (SD). Categorical variables are reported as *N* (%). Total count values may not sum to 67 due to missing data. Bolded *P* values correspond to covariates that met a level of significance of *P* < 0.2 for inclusion in the multivariable model. BMI: body mass index. cm: centimeter.

**Table 2:** Treatment characteristics.

Characteristic	Major wound complication			Any wound complication		
	Yes	No	*P*	Yes	No	*P*
*Resection type*			1.0			0.76
Primary resection	11 (79%)	43 (81%)		17 (77%)	37 (82%)	
Reresection	3 (21%)	10 (19%)		5 (23%)	8 (18%)	
*Wound closure*			**0.19**			**0.01**
Primary	6 (43%)	33 (62%)		8 (36%)	31 (69%)	
Skin graft and/or vascularized flap reconstruction	8 (57%)	20 (38%)		14 (64%)	14 (31%)	
*Preoperative chemotherapy*			1.0			0.48
Yes	4 (29%)	15 (28%)		5 (23%)	14 (31%)	
No	10 (71%)	38 (72%)		17 (77%)	31 (69%)	
*Institution providing radiation therapy*			0.6			0.39
Our institution	12 (86%)	49 (92%)		19 (87%)	42 (93%)	
Other	2 (14%)	4 (8%)		3 (13%)	3 (7%)	
*Radiation modality*			0.74			**0.008**
3D conformal	4 (29%)	13 (25%)		10 (45%)	7 (16%)	
IMRT	10 (71%)	30 (57%)		12 (55%)	28 (62%)	
Proton therapy	0 (0%)	10 (18%)		0 (0)	10 (22%)	
*Radiation timing*			0.43			0.72
Preoperative	11 (79%)	46 (87%)		18 (82%)	39 (87%)	
Postoperative	3 (21%)	7 (13%)		4 (18%)	6 (13%)	
*Use of radiation boost*			1.0			1.0
Yes	1 (8%)	5 (10%)		2 (10%)	4 (9%)	
No	12 (92%)	46 (90%)		19 (90%)	39 (91%)	
*Use of radiation bolus*			0.5			**0.16**
Yes	5 (36%)	12 (24%)		8 (38%)	9 (21%)	
No	9 (64%)	37 (76%)		13 (62%)	33 (79%)	
*Grade ≥2 radiation dermatitis*			0.51			**0.02**
Yes	5 (36%)	13 (25%)		10 (45%)	8 (19%)	
No	9 (64%)	38 (75%)		12 (55%)	35 (81%)	
*Cumulative radiation dose (gy)* ^*∗*^	5027 (410)	5015 (296)	0.85	5021 (323)	5016 (323)	0.34
*Radiation planning target volume (cc)* ^*∗*^	1646 (1526)	1314 (1663)	**0.12**	1747 (2382)	1206 (1092)	0.53

^*∗*^Continuous variables are reported as mean (SD). Categorical variables are reported as *N* (%). Total count values may not sum to 67 due to missing data. Bolded *P* values correspond to covariates that met a level of significance of *P* < 0.2 for inclusion in the multivariable model. IMRT: intensity-modulated radiation therapy. gy: gray. cc: cubic centimeters.

**Table 3:** Wound complications in study sample.

Wound type	*N* (%)
*Major wounds*	*14 (21%)*
Reoperation	12
Nonsurgical readmission	1
Prolonged deep packing	1
*Minor wounds*	*8 (12%)*
In-office debridement	5
Oral antibiotics	2
Hyperbaric oxygen	1
*Any wounds*	*22 (33%)*

**Table 4:** Multivariable analysis of predictors for any wound complications.

Predictors	Adjusted OR (95% CI)	*P*
Diabetes versus no diabetes	9.58 (1.42–64.83)	0.02
Grade ≥2 versus grade <2 radiation dermatitis	4.82 (1.20–19.21)	0.03
3D conformal RT versus IMRT or proton radiation	4.55 (1.09–20.0)	0.04

OR: odds ratio; CI: confidence interval; IMRT: intensity-modulated radiation therapy.
